# Timing rather than user traits mediates mood sampling on smartphones

**DOI:** 10.1186/s13104-017-2808-1

**Published:** 2017-09-16

**Authors:** Beryl Noë, Liam D. Turner, David E. J. Linden, Stuart M. Allen, Gregory R. Maio, Roger M. Whitaker

**Affiliations:** 1 School of Computer Science and Informatics, Cardiff University, The Parade 5, Cardiff, CF24 3AA UK; 2 School of Psychology, Cardiff University Brain Research Imaging Centre, Maindy Road, Cardiff, CF24 4HQ UK; 3 School of Medicine, MRC Centre for Neuropsychiatric Genetics and Genomics, Maindy Road, Cardiff, CF24 4HQ UK; 4 Department of Psychology, University of Bath, 10 West, Bath, BA2 7AY UK

**Keywords:** Mood, Mood sampling, Study design, Smartphone, Smartphone study, Experience sampling methodology

## Abstract

**Objective:**

Recent years have seen an increasing number of studies using smartphones to sample participants’ mood states. Moods are usually collected by asking participants for their current mood or for a recollection of their mood states over a specific period of time. The current study investigates the reasons to favour collecting mood through current or daily mood surveys and outlines design recommendations for mood sampling using smartphones based on these findings. These recommendations are also relevant to more general smartphone sampling procedures.

**Results:**

N=64 participants completed a series of surveys at the beginning and end of the study providing information such as gender, personality, or smartphone addiction score. Through a smartphone application, they reported their current mood 3 times and daily mood once per day for 8 weeks. We found that none of the examined intrinsic individual qualities had an effect on matches of current and daily mood reports. However timing played a significant role: the last followed by the first reported current mood of the day were more likely to match the daily mood. Current mood surveys should be preferred for a higher sampling accuracy, while daily mood surveys are more suitable if compliance is more important.

**Electronic supplementary material:**

The online version of this article (doi:10.1186/s13104-017-2808-1) contains supplementary material, which is available to authorized users.

## Introduction

There are numerous approaches to assessing mood (e.g. using the PANAS [1], POMS [2], BMIS surveys [3], or the experience sampling method [4]), but only relatively recently have mood surveys migrated to the smartphone [5–7]. Sampling the mood of participants in this way requires a design choice to be made: either sampling current moods several times per day or collecting only once. This choice has different implications for the participant, representing a trade-off between interruption [8] and recall [9]. A single “daily” mood report requires the participant to be accurate with their reflection of the whole day, whereas “current” mood reporting samples a participant’s mood at a particular time, but requires more frequent interruption of the user. As such, individual differences between participants and reporting circumstances could influence responses. Delespaul [10] has already highlighted the importance of not exceeding six data collection points per day for experience sampling procedures. Given the burden the response requests place on the participant, especially when they are not interruptible, it is important to establish whether daily and current mood measures are interchangeable, resulting in recommendations for different data collection frequencies.

Individual differences may result in alternative response dispositions towards surveys [11–15]. Work in this area has found associations with big five personality traits but also need for cognition [16]. While these findings were collected from online surveys, participants might be differently inclined to smartphone-based surveys. Indeed, smartphone interruption to gain user attention and response is an emerging and already complex field in its own right [8].

Specifically in relation to mood surveys, individual differences in personality [17–19], impulsivity [20], and proneness to smartphone addiction [21, 22] could contribute to a mismatch in the responses to current and daily reports. Also the intensity of current reported mood states and the amount of time between current and daily mood reports might have moderating effects, which can be predicted based on memory biases such as the recency effect [23], the primacy effect [23], and the peak-end rule [24].

## Main text

### Methods

#### Participants

Seventy-six participants were recruited through posters and online advertisement at Cardiff University UK, aged between 19 and 46 (M = 24.94, SD = 5.69). Thirty-nine participants were male, 36 female and 1 participant chose not to disclose their gender. Participants were selected on two aspects: they needed to have a smartphone running Android 4.4 or higher, and they had to have no history of mental illness.

The Android platform was both chosen for convenience (similar data collection on iOS is impeded by the operating system) and to reach a larger number of participants (at the start of the study, in May 2016, 46% of the British population uses Android and 43.39% iOS ) [25, 26]. Participants were also selected on absence of mental illnesses. This was done so mental illnesses, especially those have affective symptoms would not become a confounding factor to this study.

#### Study design

All participants attended a briefing session where they downloaded our custom made application “Tymer”, were given instructions on how to use the app and the distinctions between the different reporting options, and were asked to complete five surveys: SAS [27], PANAS [1], BFI [28], MCQ [29], and a demographics and smartphone use questionnaire. After 8 weeks of using Tymer, participants returned for a debriefing session where they retook the surveys and received monetary compensation.

The Tymer application prompted participants to report their current mood (CM) using a dartboard-shaped interface (as shown in Additional file 1: Figure S1 (left)), based on the circumplex model of affect [30], up to three times per day. Notifications requesting the user to complete CM reports only arrived while the smartphone was in use to maximise the likelihood of response. Additionally, participants were asked to select their daily mood (DM) (see Additional file 1: Figure S1 (right)), as part of an evening survey that was sent on the first screen unlock after 19:00 every day. Both type of reports could also be completed through the application interface. Notification expiration time was set to 10 min for CM prompts and at 23:55 for the DM survey. A typical day using the Tymer application is depicted in Additional file 1: Figure S2.

#### Data cleaning

While 76 participants were recruited, smartphone data was only obtained from 64 participants due to hardware problems and withdrawals from participation. The number of completed and uncompleted reports are shown for both types of surveys in Table 1. In 8 weeks, participants should have completed 56 DM surveys and 168 CM surveys. The mean participation rate considering these numbers was 79.8% for DM and 80.6% for CM surveys.

**Table 1:** Frequencies of completion and source of CM and DM surveys

		CM		DM	
		Count	%	Count	%
All		14208	100	4416	100
*Source*					
Notification		13399	94.31	3851	93.56
App interface		809	5.69	265	6.44
*Completion*					
Expired		4468	31.45	154	3.74
Abandoned		64	0.45	178	4.32
Completed		8676	61.06	3266	79.35
Dismissed		1000	7.04	518	12.59
Blank		1	0.01	0	0

Pairs of CM and DM surveys undertaken on the same day were analysed. In case several DM surveys were completed for 1 day, only the first one was considered. This resulted in 7893 unique CM and 2667 unique DM surveys being analysed, resulting in 7893 pairs of current and daily mood surveys. In total, there were 1835 instances where a day had at least one CM-DM match, this represents 68.80% of the 2667 reported DM (also see Additional file 2). The BFI was mistakenly done twice by one participant at briefing; only the first submission was used.

#### Comparison of proportion of matches/non-matches to random

A binomial test was used to compare the proportion of matches and non-matches between CM and DM responses against the number of such matches that would occur in a random sample (1/9 = 11%). The proportion of matches was statistically greater than 11% ($p < .001$) with 2529 (32.04%) of the CM and DM survey pairs reporting the same mood.

#### Data transformation

Since participation was voluntary, each participant had a varying number of data points. To summarise the data per participant, it was modified by either adopting the count (number of matching or non-matching CM and DM survey pairs), the median (time difference between CM and DM surveys, intensity of the current mood reports) or calculating a percentage (number of matching CM-DM pairs per day) of all instances concerning a participant. Spearman’s correlation, Wilcoxon-Mann-Whitney test, and Wilcoxon signed ranks test can be applied due to these transformations.

### Results

#### Effect of time on CM-DM report matches

**Fig. 1:**
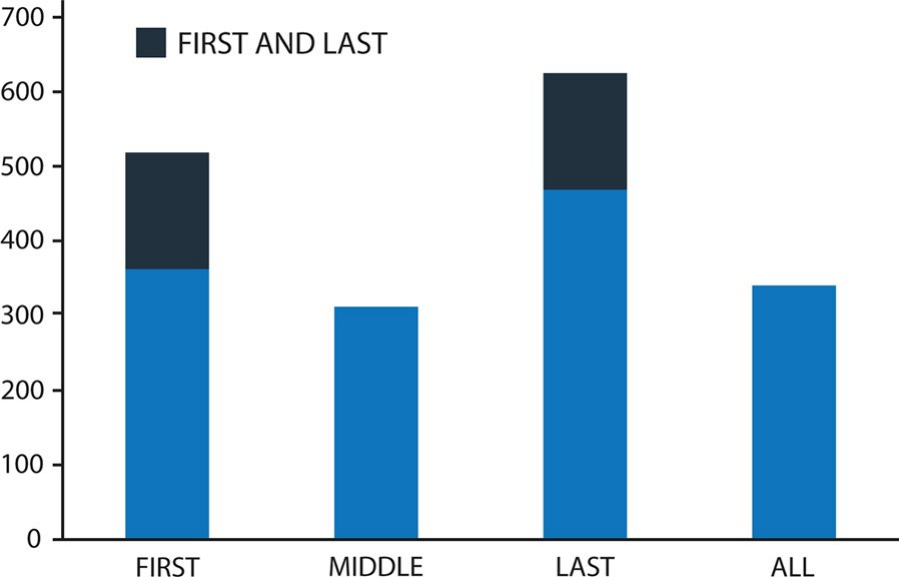
Number of reports where the CM and DM match

The median time between evening and current surveys was significantly shorter for matches than non-matches (*Z* = −3.103, *p* = .002). For each participant, days in which matches in mood response occurred were categorised as follows: ALL, where all reported CM(s) of the day matched the reported DM, FIRST, LAST and MIDDLE where the reported CM(s) of the day that matched the reported DM were respectively the first, last, or neither first nor last (see Additional file 3). Since days that had both matches for the first and last reported CM would fall into both of these categories, they were split evenly between them (see Fig. 1). The resultant categorisation was therefore mutually exclusive. It should also be noted, that, since a day was defined as starting at 00:00 and ending at 23:59, some matches could have occurred after the evening survey was completed.

Wilcoxon’s signed rank test was used to compare the count of all categories to one another. Matches in the LAST category were found to be significantly more frequent ($p < .01$) than in all other categories ($M = 8.24$, $SD = 5.44$); followed by matches in the FIRST category ($M = 6.57$, $SD = 4.49$), which were statistically greater ($p < .01$) than the ALL ($M = 5.31$, $SD = 4.40$) and MIDDLE ($M = 1.89$, $SD=3.12$) categories. These results and their medium to high effect sizes are shown in Table 2 [31].

**Table 2:** Z values and effect sizes for each category pair

Z values					
		FIRST	ALL	MIDDLE	LAST
FIRST		–	−2.664**	−2.875**	−2.730**
ALL			–	−.140	−4.103***
MIDDLE				–	−4.748***
LAST					–

Effect sizes					
		FIRST	ALL	MIDDLE	LAST
FIRST		–	.333	.359	.341
ALL			–		.513
MIDDLE				–	.594
LAST					–

*** p <.001, ** p <.01, * p <.05

Additional results can be found in Additional file 4.

### Discussion

This study has shown that there is evidence to suggest that CM and DM reports are interchangeable as a methodology to sample participant mood. Indeed 68.8% of the recorded DM matched a CM that was reported in the same day. None of the investigated intrinsic characteristics (gender, age, personality, etc.) had an effect on matches of current and daily moods, suggesting that a specific participant sample would not justify the choice of one over the other reporting method.

Further results show that time intervals between CM and the DM survey had a significant effect on CM-DM matches. This could imply that daily mood does not reflect as much the entirety of the day as intended. As predicted by memory biases, the last reported CM reports were more likely to match the DM report due to them being closer in time, while the first reported CM report came in second in terms of similarity. These findings are consistent with reports of the serial position effect [23], which shows a higher probability of recall for initial and final elements from a list, with lower probability for elements in-between, and with the final element having the highest probability overall. This implies that CM reports might be more accurate to sample current mood than DM reports are for collecting daily mood since memory biases come into play that slightly hinder the formation of an accurate daily summary. These findings were supported by medium and high effect sizes (r > .333), which show that the sample size was sufficient to find these effects.

Daily mood surveys were also at a disadvantage considering the number of dismissed notifications (12.59% vs 7.04% for CM surveys), while its percentage of survey completions via the app interface is similar to that of CM surveys (6.44 and 5.69% respectively). However, participants might have not needed to dismiss notifications for CM as they expired more quickly. CM surveys were more invasive as participants were prompted at least three times per day, while DM surveys only happened once at set time. This is likely to have contributed to an overall higher completion rate for DM (79.35%) than CM (61.06%) reports.

Our average completion rates (about 80% for both types of surveys) were quite high considering the length of our study and are mostly higher than those reported in similar studies [32]. We believe the best way to increase compliance and accuracy of participants, would be to increase the incentives for good performance through feedback (e.g. higher rewards by providing visualisation of historic personal data or gamifying parts of the app). While feedback has been shown to increase compliance [33], the increased awareness could however influence the participant. While Downes-LeGuin and colleagues [34] have shown gamification to be ineffective to increase engagement even though it increased satisfaction, other studies do report heightened engagement [35].

Additional discussion points can be found in Additional file 5.

### Conclusions

Whether current or daily mood surveys should be used to collect affective data on participants highly depends on the requirements of the study, and whether related *in-situation* context or device usage is important. One also needs to consider what exactly needs to collected: momentary mood fluctuations, or prevailing mood of the day. However our results indicate that both approaches can be used with confidence, albeit noting specific implications for each.

If participant compliance is of high importance, daily surveys should be favoured as participants are more likely to dismiss notifications if they are frequent or come at inopportune moments.

We note that while the investigated intrinsic characteristics did not affect the two surveys differently, effects for time did come into play. Current mood surveys are more accurate as the participant is directly asked for the mood state they are in at that instant, while a daily mood survey requires the participant to provide a summary of the moods they have felt during the day and this cognitive task is vulnerable to memory biases.

## Limitations

This study had a few limitations:

- Only Android users were selected. This has consequences on the generalisability of our results since previous literature has shown that Android and iPhone user groups may be quite distinct [36].
- CM and DM were collected simultaneously and could have influenced each other.
- Since the mood measures were all self-reported, given responses could be dishonest or not well-estimated. Misclicks can also occur.
- Smartphone data was missing from 12 participants.

## Additional files

**Additional file 1.** Study design. Additional information on demographics and smartphone use questionnaire and two additional figures.

**Additional file 2.** Additional data. One additional table and three additional figures.

**Additional file 3.** Classification example. Additional figure.

**Additional file 4.** Additional results.

**Additional file 5.** Discussion of further applications of the Tymer app.

## Abbreviations

CM: current mood
DM: daily mood
