# Correction to: Timing rather than user traits mediates mood sampling on smartphones

**DOI:** 10.1186/s13104-020-05108-z

**Published:** 2020-05-28

**Authors:** Beryl Noë, Liam D. Turner, David E. J. Linden, Stuart M. Allen, Gregory R. Maio, Roger M. Whitaker

**Affiliations:** 1 School of Computer Science and Informatics, Cardiff University, The Parade 5, Cardiff, CF24 3AA UK; 2 School of Psychology, Cardiff University Brain Research Imaging Centre, Maindy Road, Cardiff, CF24 4HQ UK; 3 School of Medicine, MRC Centre for Neuropsychiatric Genetics and Genomics, Maindy Road, Cardiff, CF24 4HQ UK; 4 Department of Psychology, University of Bath, 10 West, Bath, BA2 7AY UK

## Correction to: BMC Res Notes (2017) 10:481 10.1186/s13104-017-2808-1

In the original article [1] an author’s name was inadvertently omitted from reference no. 7.

Khue LM, Jarzabek S. Demonstration paper: mood self–assessment on smartphones. In: Proceedings of the conference on wireless health. ACM, New York, NY, USA. 2015. 10.1145/2811780.2811921.

should therefore be corrected as follows:

Khue LM, Ouh EL, Jarzabek S. Demonstration paper: mood self–assessment on smartphones. In: Proceedings of the conference on wireless health. ACM: New York; 2015. 10.1145/2811780.2811921.
