# Supplementary material for: Timing rather than user traits mediates mood sampling on smartphones
Source: BMC Res Notes. 2017 Sep 16;10:481. doi: 10.1186/s13104-017-2808-1 (PMC5602857; doi:10.1186/s13104-017-2808-1)

Additional File 1: Study design

The demographics and smartphone use questionnaire was derived from a standard demographics and health questionnaire used in the School of Psychology, Cardiff University. A specific question about smartphone handedness was added as responses could vary from the regular handedness question and this variable could be considered a confounding factor when participants answered surveys on their phone (due to proximity of their fingers to response options on screen). Furthermore, participants were asked a few questions on their perceived smartphone use to provide a personal context.

Figure 1 Screenshots of the CM (left) and DM (right) surveys in Tymer. In these examples, the user has selected his current mood to be "moderately relaxed" and has not yet chosen a daily mood.

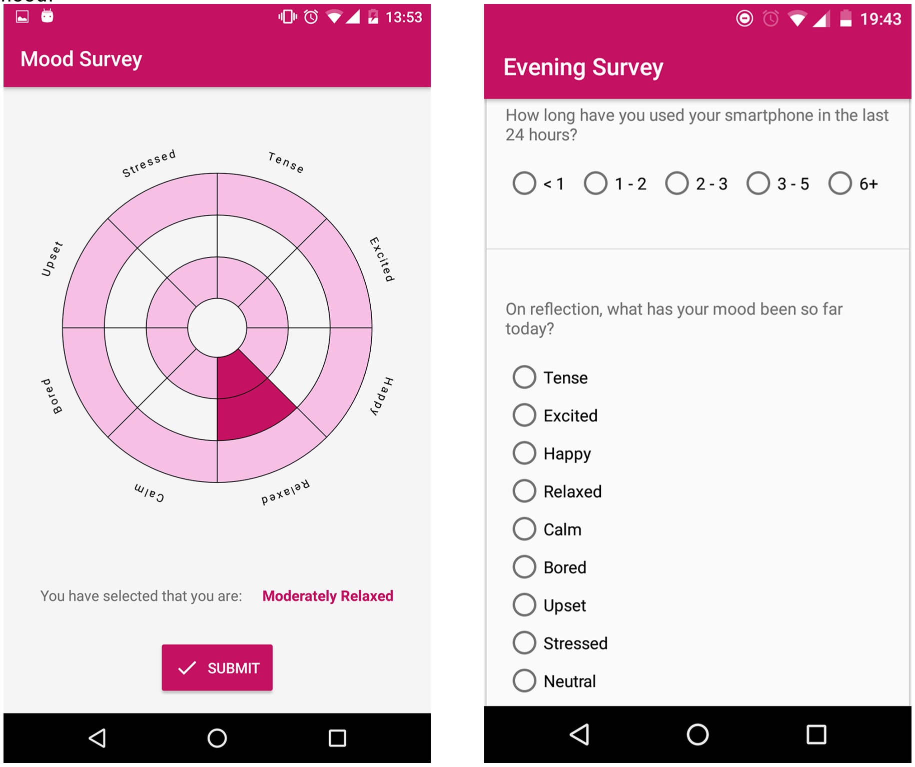

Figure 2 Example of a day using the Tymer app.

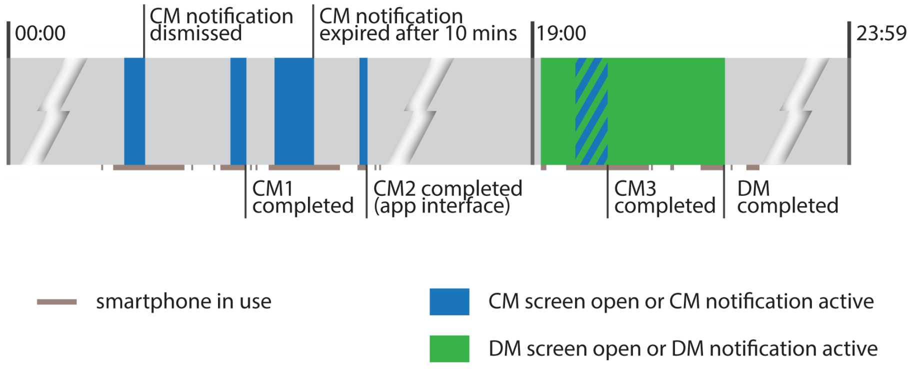

Supplement: Supplementary file 1 — Additional file 1. Study design. Additional information on demographics and smartphone use questionnaire and two additional figures. [file 13104_2017_2808_MOESM1_ESM.pdf]
