# Supplementary material for: Timing rather than user traits mediates mood sampling on smartphones
Source: BMC Res Notes. 2017 Sep 16;10:481. doi: 10.1186/s13104-017-2808-1 (PMC5602857; doi:10.1186/s13104-017-2808-1)

Additional File 2: Additional data

Table 1 Frequencies of DM that had at least one CM match.

mood	count	percentage
neutral	93	5.07
tense	92	5.01
excited	93	5.07
happy	455	24.80
relaxed	569	31.01
calm	219	11.93
bored	146	7.96
upset	59	3.22
stressed	109	5.94

Figure 1 Count of reported moods for CM and DM surveys.

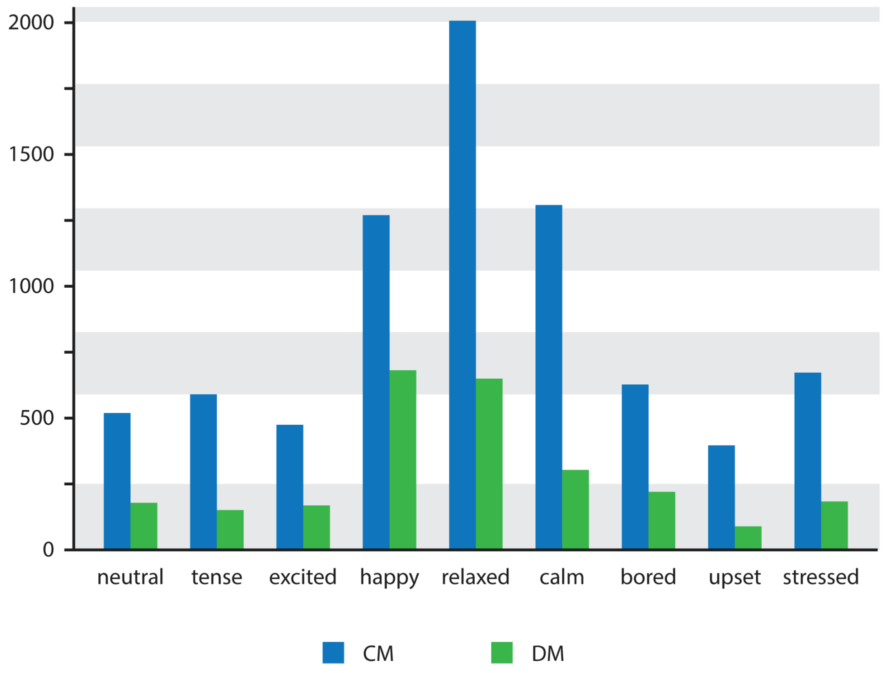

Figure 2 Percentages of reported moods for CM and DM surveys.

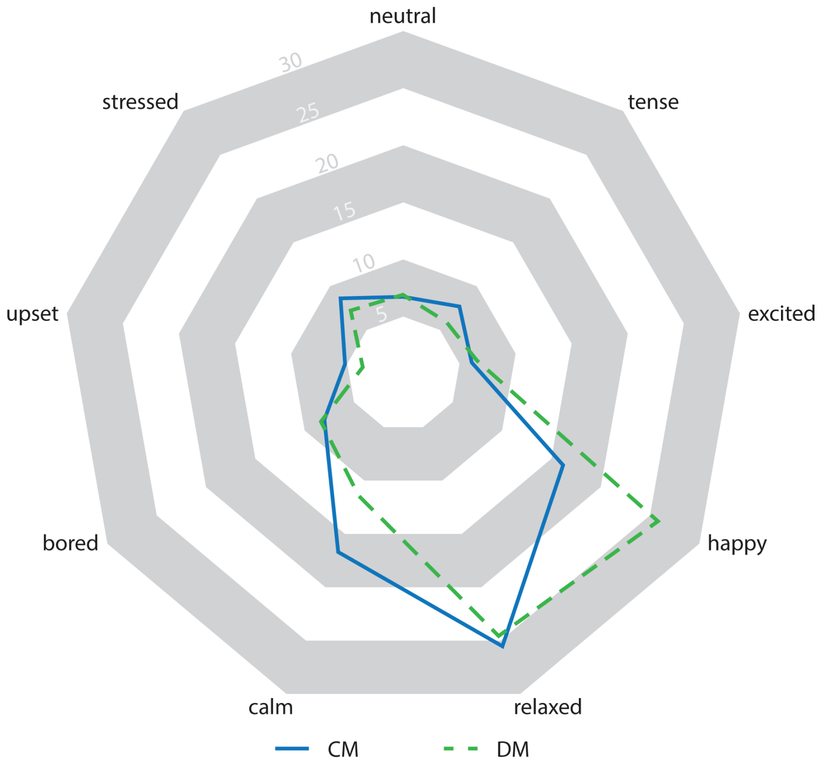

Figure 3 Count of reported intensities for CM surveys.

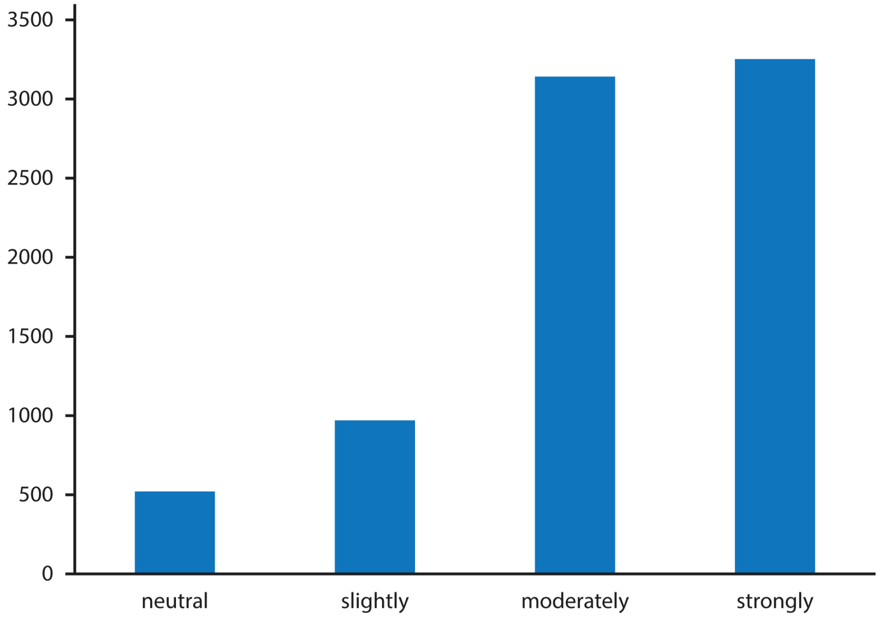

Supplement: Supplementary file 2 — Additional file 2. Additional data. One additional table and three additional figures. [file 13104_2017_2808_MOESM2_ESM.pdf]
