# Supplementary material for: Timing rather than user traits mediates mood sampling on smartphones
Source: BMC Res Notes. 2017 Sep 16;10:481. doi: 10.1186/s13104-017-2808-1 (PMC5602857; doi:10.1186/s13104-017-2808-1)

Additional File 3 - Classification example

Figure 1 Examples of days that have at least one CM-DM match and what they would be classified as. The same colour represents the same reported mood.

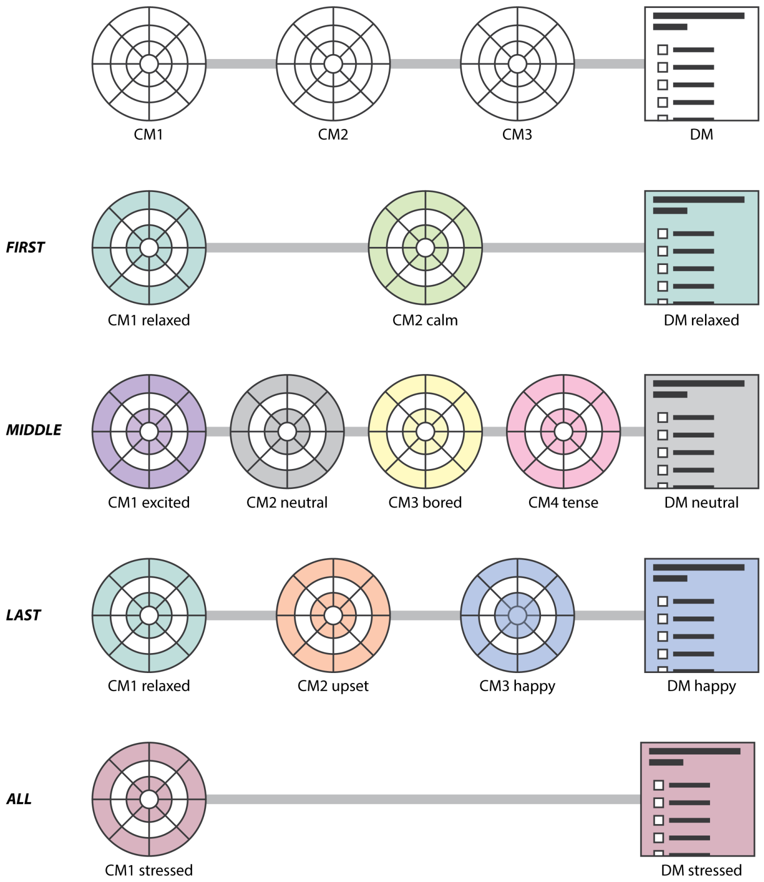

Supplement: Supplementary file 3 — Additional file 3. Classification example. Additional figure. [file 13104_2017_2808_MOESM3_ESM.pdf]
