# Supplementary material for: Timing rather than user traits mediates mood sampling on smartphones
Source: BMC Res Notes. 2017 Sep 16;10:481. doi: 10.1186/s13104-017-2808-1 (PMC5602857; doi:10.1186/s13104-017-2808-1)
Supplement: Supplementary file 4 — Additional file 4. Additional results. [file 13104_2017_2808_MOESM4_ESM.pdf]

Additional File 4: Additional results

Results

Effects of individual and intrinsic characteristics

The percentage of matching CM-DM pairs per day from the total CM-DM pairs was identified per person (from now on named PM) and compared to that person's score on a variety of surveys using Spearman's correlation. PM was found not to be correlated to any of the personality traits: openness (r_s = -.420, p = .744), conscientiousness (r_s = .060, p = .636), extraversion (r_s = .085, p = .503), agreeableness (r_s = .059, p = .641), and neuroticism (r_s = -.189, p = .153). PM was also not correlated to the impulsivity score given by the MCQ (r_s = -.039, p = .760) and to the smartphone addiction score [1] for both the briefing (r_s = -.100, p = .430) and debriefing (r_s = -.024, p = .851) sessions.

Lastly, the effect of demographic variables and PM were analysed using the Wilcoxon-Mann-Whitney test, Spearman's correlation and Kruskal-Wallis tests. No statistically significant difference was found for gender (Z = -1.729, p = .084), age (r_s = .175, p = .167), bilingualism (Z = -.840, p = .401), participant's first language ($\chi^2(12) = 17.754$, p = .123), employment status ($\chi^2(6) = 4.639$, p = .591), education level ($\chi^2(6) = 8.839$, p = .183), handedness ($\chi^2(1)$ = .013, p = .910), handedness during smartphone use ($\chi^2(2) = 4.346$,. p = .114) and estimated smartphone usage in relation to population average ($\chi^2(2) = 5.476$, p = .065).

Effects of mood report characteristics

The intensity of reported CMs and the time interval between CM and DM were investigated using Wilcoxon signed-ranks tests. The intensity difference between matches and non-matches was not statistically significant (Z = -1.726, p = .084), but the median time between evening and current surveys was significantly shorter for matches than non-matches (Z = -3.103, p = .002). This result has a medium effect size of .388, calculated using Rosenthal (1994)'s $r = \frac{Z}{\sqrt{N}}$ formula [2].

References

1. Kwon, M., Lee, J.Y., Won, W.Y., Park, J.W., Min, J.A., Hahn, C., Gu, X., Choi, J.H., Kim, D.J.: Development and Validation of a Smartphone Addiction Scale (SAS). PLoS ONE **8**(2) (2013). doi:10.1371/journal.pone.0056936
2. Rosenthal, R.: Parametric measures of effect size. In: Cooper, H.M. (ed.) The Handbook of Research Synthesis, pp. 231–244. Russell Sage Foundation, New York (1994)
