# Supplementary material for: Timing rather than user traits mediates mood sampling on smartphones
Source: BMC Res Notes. 2017 Sep 16;10:481. doi: 10.1186/s13104-017-2808-1 (PMC5602857; doi:10.1186/s13104-017-2808-1)
Supplement: Supplementary file 5 — Additional file 5. Discussion of further applications of the Tymer app. [file 13104_2017_2808_MOESM5_ESM.pdf]

Additional File 5 - Discussion of further applications of the Tymer app

Discussion

The Tymer app has potential for further applications beyond this study, as the easy to use visual interface for mood reports and short questionnaires can easily be modified for other studies, especially in the mental health domain. Using different mood options and having the possibility to select different mood intensities allows for detailed categorisation of mood states. This requires users to label their affective states accurately. Swinkels and Guiliano (1995) [1] have shown that mood labelling was associated with positive affect and higher self-esteem, while mood monitoring alone predicted negative affect and rumination on negative mood. In the future, the app could also be used to deliver interventions should increased and/or prolonged negative mood reports raise concerns, possibly through use in partnership with a health professional. However, before any use in clinical monitoring, formal validation studies against established clinical measures (such as clinical questionnaires and symptoms scales) would be needed.

References

1. Swinkels, A., Guiliano, T.A.: The measurement and conceptualization of mood awareness: Monitoring and labeling one's mood states. Personality and social psychology bulletin **21**(9), 934–949 (1995)
